# COVID-19 Vaccine Hesitancy Among Health Care Workers in Thailand: The Comparative Results of Two Cross-Sectional Online Surveys Before and After Vaccine Availability

**DOI:** 10.3389/fpubh.2022.834545

**Published:** 2022-08-01

**Authors:** Chatkamol Pheerapanyawaranun, Yi Wang, Nachawish Kittibovorndit, Nopphadol Pimsarn, Kanchanok Sirison, Yot Teerawattananon, Wanrudee Isaranuwatchai

**Affiliations:** ^1^Health Intervention and Technology Assessment Program (HITAP), Department of Health, Ministry of Public Health, Nonthaburi, Thailand; ^2^Saw Swee Hock School of Public Health, National University of Singapore, Singapore, Singapore; ^3^Institute of Health Policy, Management and Evaluation (IHPME), University of Toronto, Toronto, ON, Canada

**Keywords:** health care workers, COVID-19, COVID-19 vaccine, vaccine, vaccine acceptance, vaccine hesitancy, Thailand

## Abstract

**Introduction:**

The arrival of COVID-19 vaccines in Thailand has supported the fight against the COVID-19 pandemic. This study examined COVID-19 vaccine acceptance among health care workers (HCWs) in Thailand before and after vaccines' availability and investigated factors (both enablers and barriers) affecting their decisions.

**Methods:**

Two online self-administered questionnaires were distributed to HCWs in two time-periods: (1) the pre-vaccine arrival period (prior to COVID-19 vaccines' arrival in Thailand, January 28 to February 16, 2021); and (2) the post-vaccine arrival period (April 21 to May 9, 2021). Descriptive analyses and multinomial logistic regression were conducted to examine factors associated with vaccine hesitancy.

**Results:**

There were 55,068 respondents in the pre-vaccine arrival period and 27,319 respondents in the post-vaccine arrival period. In the pre-vaccine arrival period, 55.0% of respondents were willing to accept the vaccines, 35.4% were uncertain, and 9.6% declined. In the post-vaccine arrival period, ~16% already received two doses of either the Sinovac or AstraZeneca vaccine, and 43% were administered one dose. Approximately 12% of those who had received the first dose were uncertain or not willing to accept the second dose. Demographic and socio-demographic factors of participants, including their sex, place of residence, and whether they were frontline COVID-19 workers, were found to be the significant factors explaining vaccination hesitancy. Moreover, when comparing the pre-vaccine arrival and post-vaccine arrival periods, it was found that older HCWs were more likely to decline a COVID-19 vaccine in the pre-vaccine arrival period; on the other hand, older HCWs were less likely to decline or be uncertain to receive a COVID-19 vaccine in the post-vaccine arrival period.

**Conclusion:**

Information on HCWs' acceptance of COVID-19 vaccines, including who is more likely to accept the vaccines, could assist in planning vaccine allocation to both HCWs and the general public, who often believe HCWs' recommendations. This study's findings set out how policies can be addressed to reduce vaccine hesitancy. This study also highlights HCWs' characteristics (including gender, work region, occupation, and history of receiving influenza vaccination) and the reasons they cited for their vaccine acceptance or hesitance.

## Introduction

The impact of the coronavirus or SARS-CoV-2 (COVID-19) pandemic is evident across various sectors, populations, and countries (1). Since the end of 2020, COVID-19 vaccines have become one of the key strategies to fight against this pandemic. For vaccines to be effective in curbing an outbreak, they need to be administered to a certain percentage of the population for the attainment of herd immunity. Further, it has been found that two doses are required for most vaccinations against many communicable diseases, including COVID-19 (2).

In 2019, the World Health Organization (WHO) reported vaccine hesitancy to be one of the top 10 global health threats to monitor (3). Although that finding was not with specific reference to COVID-19, vaccine hesitancy for COVID-19 vaccines is now an immediate threat to the successful administration of COVID-19 vaccinations throughout the world (4). The acceptance of COVID-19 vaccines varies across populations and countries depending on factors such as vaccine prioritization groups and individual beliefs. Although the number of partially and fully vaccinated people are increasing (5), there is evidence indicating that some people who were administered one dose have missed their deadline for receiving the second dose, thereby highlighting the ground realities of vaccine hesitancy (6).

As recommended by the Strategic Advisory Group of Experts on Immunization's Values Framework for the allocation and prioritization of the COVID-19 vaccination (7), and the Roadmap for prioritizing population groups for vaccines against COVID-19 (8), health care workers (HCWs) have been prioritized to receive the COVID-19 vaccination because of several reasons. One of the key reasons is that they are at higher risk of virus exposure. Being one of the first groups to receive (often innovative) vaccines, vaccine hesitancy among health professionals is not an uncommon problem (9). Additionally, HCWs represent an essential group of members in communities and their voices are given importance in relation to health-related issues (10). Therefore, understanding potential factors influencing HCWs' acceptance of COVID-19 vaccines can assist in planning interventions and strategies for vaccine distribution to the general public.

Using a health behavior model as a guiding framework for identifying factors that influence health-related behavior, it has been found that several factors may influence vaccine hesitancy for COVID-19 vaccines (11). Given the importance of vaccine hesitancy in the combat against the COVID-19 pandemic, there have been a number of studies examining vaccine hesitancy in various countries and across populations (12–15). Nevertheless, contextualization is of relevance to vaccine priority groups and for vaccine hesitancy. The number of studies on vaccine hesitancy in low- and middle-income countries (LMICs) settings are limited, and Thailand could be considered a case study of an upper middle-income country in Asia. Moreover, information on how vaccine acceptance may change over time, such as before and after vaccines' arrival, can lead to a better understanding of changing trends on vaccine hesitancy. The planning of vaccine administration, especially in a sudden pandemic scenario, is usually based on information collected before vaccines' availability. As Thailand was preparing for the arrival of COVID-19 vaccine in early 2021, the COVID-19 Vaccine Administration Working Group established by the Ministry of Public Health in Thailand requested information to support their vaccination dissemination plan recognizing that vaccine hesitancy has been one of the top global health threats even before COVID-19. Therefore, this study was commissioned by the Thai government to provide information on whether vaccine hesitancy was a concern among healthcare workers in Thailand and whether preferences prior to vaccines' availability may differ with actual preferences (once the vaccines became available). The study's findings may also be helpful for future vaccine planning, for example, which groups among healthcare workers may be more likely to have COVID-19 booster vaccine hesitancy. By focusing on the important issue of vaccine hesitancy among HCWs in a unique setting over time, this study examines COVID-19 vaccine hesitancy among HCWs in Thailand before and after vaccines' availability, and to survey factors (both enablers and barriers) affecting their decisions.

## Methods

### Study Design

Data were collected through an online self-administered questionnaire (on the SurveySparrow platform) distributed through various channels in two time periods: (1) the pre-vaccine arrival period (prior to the COVID-19 vaccinations' arrival in Thailand, January 28 to February 16, 2021); and (2) the post-vaccine arrival period (after the vaccines became available, April 21 to May 9, 2021). However, it is to be noted that the starting date of COVID-19 vaccination in Thailand for HCWs was on Feb 28, 2021 (16).

The questionnaire was drafted based on a literature search on the topic of vaccine acceptance. Opinions of subject matter experts, who were members of the Vaccine Academic Working Group at the Thai Ministry of Public Health (MOPH), were used in the design stage to refine the survey questions during their consultation meeting. Subsequently, the questionnaire was validated in a pilot test among health professionals and researchers (*N*~100) and was updated based on their feedback to improve clarity and comprehensibility of the questions. The total number of questions was 25 for the pre-vaccine arrival period, and 28 for the post-vaccine arrival period (as there were three additional questions incorporated). A copy of the final questionnaire is presented in Supplementary Material 1.

### Participants, Setting, and Recruitment

The target population were HCWs in Thailand. Inclusion criteria included: (1) participants who were ≥ 17 years old (at the time of the survey); (2) participants who work as healthcare workers including, but not limited to, doctors, dentists, pharmacist, nurses, medical laboratory workers, patient aids, village health volunteers and migrant health volunteers, public health officers, and others who are working in both public and private health facilities; (3) participants who have not received any COVID-19 vaccines before at the time of enrollment (only pre-vaccine arrival period); and (4) Sample who were able to understand Thai written and provide information with an electronic device. Exclusion criteria were: (1) participants who did not provide consent; (2) participants <17 years old; (3) participants who were not healthcare workers; and (4) participants who received any COVID-19 vaccines before at the time of enrollment (only pre-vaccine arrival period).

In the pre-vaccine arrival period, there were 55,068 respondents, and, in the post-vaccine arrival period, there were 27,319 respondents. Descriptive analyses were conducted separately for each time period, and data from the two time periods were combined for a multinomial logistic regression analysis. Written consent, which was provided online, was obtained from each participant before the commencement of the COVID-19-vaccination-related questions in the survey. The survey was voluntary. Only non-identifiable data were collected, and the findings are presented in an aggregate manner.

Several strategies and channels were used to distribute the survey in both periods. Official letters signed by the Permanent Secretary of the Thai MOPH were sent to all 77 Provincial Health Offices including the University Hospital Network (UHosNet), the Private Hospital Association, and other relevant networks. The online survey link was shared with media channels (both online and through cable television). Additionally, social media channels, that is Twitter, LINE, and Facebook, were used to disseminate the online survey. Further, the snowball sampling method was utilized to increase the number of respondents.

### Variables

The questionnaire has five sections: (1) demographics characteristics; (2) socio-economic factors; (3) health-related factors; (4) perceived risk of COVID-19; and (5) perceived enablers and barriers of COVID-19 vaccination.

The main purpose of the survey was regarding the COVID-19 vaccine, with 3 potential answer options (yes, no, uncertain) to the question “Would you be willing to receive the COVID-19 vaccine?”

#### Demographic Characteristics (Four Questions)

This section included questions on age (as a continuous variable), sex (male, female, others), marital status [single, married, and others (separated, widowed, or divorced)], and religion (Buddhism and others).

#### Socio-Economic Factors (Six Questions)

A 5-item section aimed to capture participants' socio-economic factors, specifically their occupation (doctors, dentists, pharmacist, nurses, medical laboratories, patient aids, village health volunteers and migrant health volunteers, public health officers, and others), and the provinces they worked in (given that Thailand has 77 provinces), which were categorized into five regions, namely Bangkok, Central, North, Northeast, and South. Furthermore, participants were asked to provide information on their workplace (urban, rural), whether they had more than one workplace (yes, no), whether they were frontline COVID-19 workers (yes, no), and the type of workplace in which they spent the majority of their time (i.e., public health centers, community hospitals, general hospitals, specialized hospitals, teaching hospitals, district/provincial health offices, the Ministry of Public Health, private hospitals/clinics, pharmacies, other health facilities, and retired or no longer working).

#### Health-Related Factors (Three Questions)

Participants were asked about their health, specifically whether they had any health conditions (e.g., diabetes, cardiovascular diseases, kidney diseases, cancer, obesity, respiratory diseases, and others). Moreover, the history of participants' influenza vaccination (had, never had, uncertain) and COVID-19 infection (had, never had, uncertain) were collected.

#### Perceived Risk for COVID-19 (Four Questions)

This section captured the perceived risk of COVID-19 by participants. Participants were asked whether they had direct contact in the screening of individuals at risk for COVID-19 or the caring of COVID-19 patients (yes, no). Their perceived risk of COVID-19 was further assessed on a six-point Likert scale (0 = definitely no to 6 = definitely yes), and it was assessed whether the participants lived with any elderly persons (aged 60 years or older) and/or individuals with chronic diseases (diabetes mellitus, hypertension, stroke, coronary artery disease, cancer with chemotherapy treatment, chronic obstructive pulmonary disease, stage 5 kidney disease, obesity or BMI ≥ 25 kg/m^2^). As elderly or people with chronic conditions are at higher risk of COVID-19, living with these groups may increase their risk for COVID-19 infection, and subsequently influence HCWs' decision to accept COVID-19 vaccines (17). Participants were also asked about the location they perceived to have highest risk of COVID-19 infection (home, workplace, community).

#### Perceived Enablers and Barriers of COVID-19 Vaccination (Five Questions)

Participants' perceived enablers and barriers were explored. First, they were asked under which condition(s) they would accept the vaccine: (1) if they received the vaccine they prefer (based on the list of existing vaccines regardless of availability in Thailand); or (2) if a certain number of people were vaccinated in the world without serious adverse events (reaction in any untoward medical event, e.g., death, life-threatening, prolonged or permanent signs, disability, or congenital disorder). Additionally, participants were asked to select the top three enablers and top three barriers to receiving a COVID-19 vaccine. Examples of enablers included approval from the World Health Organization (WHO) or Thai Food and Drug Administration, seeing the country's leader, family, or friends getting vaccinated, belief that the vaccines can prevent infection, or living in high-risk areas. Examples of barriers included concerns regarding vaccine efficacy, concerns of short-term and/or long-term side effects, belief that they were not at risk, or travel restrictions. Furthermore, participants commented on the safety of COVID-19 vaccines compared to the influenza vaccine (less than, equal to, greater than, or uncertain). Lastly, participants were asked, as HCWs, whether they would make recommendations to others to receive a COVID-19 vaccine.

### Data Analysis

Descriptive analyses were conducted to provide general information about the respondents in each time period and overall, where continuous variables were reported as mean, standard deviation (SD), minimum and maximum, and categorical variables were reported as absolute (numbers) and relative (percentage) frequencies.

The findings on enablers and barriers to receive COVID-19 vaccines were reported in both descriptive and explanatory manners. The top three enablers and barriers were ranked based on number of times selected. In addition, a multinomial logistic regression was conducted to identify factors associated with vaccine hesitancy by reporting adjusted relative risk ratios (RRRs) of those not willing to accept a vaccine or were uncertain compared to those willing to accept a vaccine. This regression model was used to address the question, “Among those waiting for COVID-19 vaccine, what factors are associated with vaccine hesitancy (comparing who decline or are uncertain about getting the vaccine to those willing to get vaccine)?” Through literature search and consultation with experts, potential explanatory variables were entered into a multinomial logistic regression model with a forward selection approach. Regression diagnostic tests were conducted. The models were checked for collinearity and homoscedasticity, and robust standard errors were used. Data were analyzed using STATA, version 16.0 (STATA Corp, College Station, TX, USA) with a statistical significance of *p* ≤ 0.05.

## Results

In the pre-vaccine arrival period, there were initially 57,695 respondents, with 56,137 (97%) respondents providing their consent. There were 1,069 observations with missing data, leading to the total sample for this period to be 55,068 (95%). In the post-vaccine arrival period, there were 27,889 respondents, with 27,395 (98%) providing their consent. There were 76 observations with missing data, leading to the total sample of 27,319 (98%). The total samples combined amounted to 82,387 respondents. The following sections report the following: (1) Vaccine hesitancy overview before and after vaccine availability; (2) Demographic characteristics, socio-economic factors, and health-related factors; (3) Perceived risk for COVID-19; (4) Perceived enablers and barriers of COVID-19 vaccination, and conditions to support COVID-19 vaccine hesitancy; (5) Factors associated with COVID-19 vaccine hesitancy; and (6) COVID-19 vaccine hesitancy after vaccines became available.

### Vaccine Hesitancy Overview Before and After Vaccine Availability

**Figure 1:**
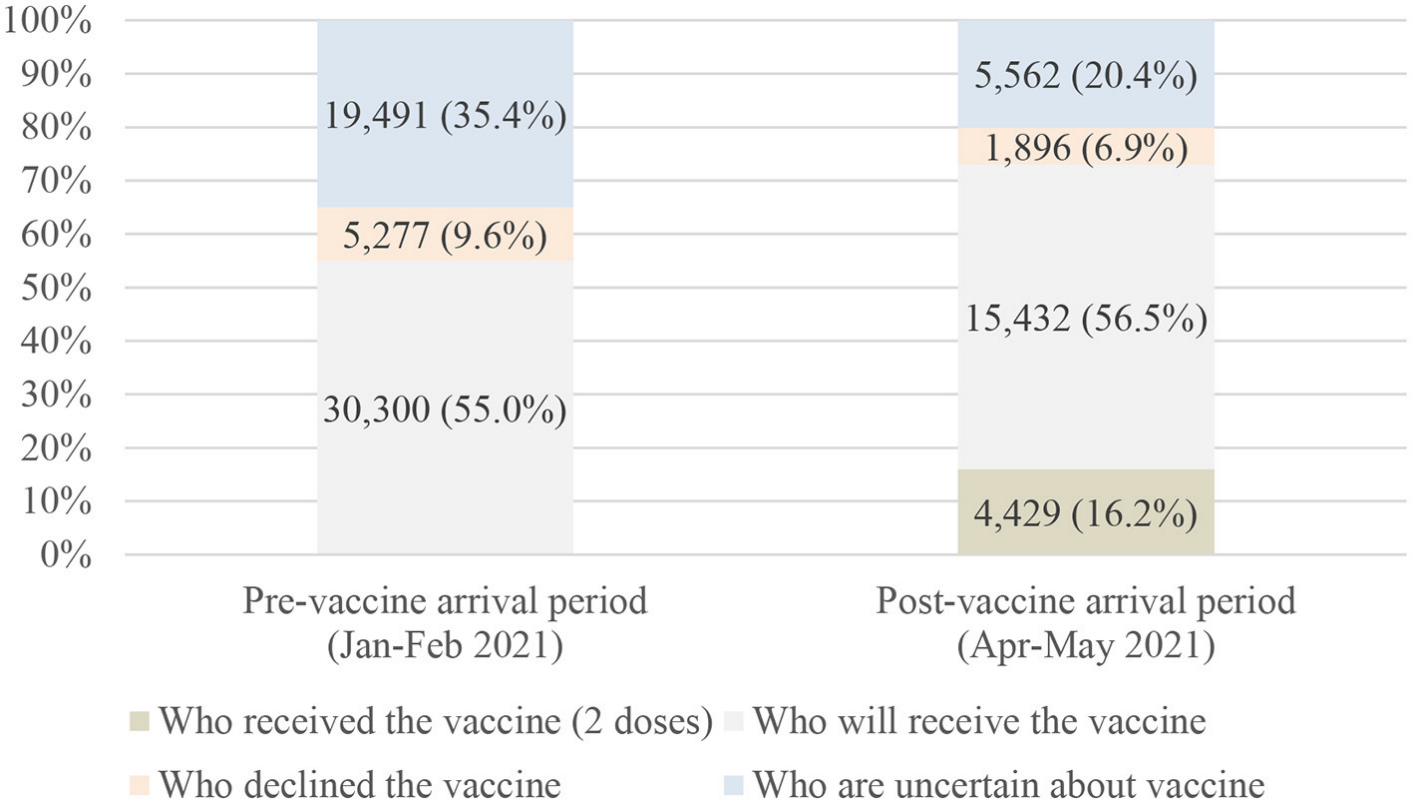
Vaccine hesitancy before and after vaccine availability (*N* = 82,387).

In the pre-vaccine arrival period, ~55% of HCWs were willing to accept either the Sinovac or AstraZeneca vaccine (vaccine brands available during the study period), whereas 35.4% were uncertain, and 9.6% declined. After the vaccines became available, the vaccine hesitancy (no or uncertain to obtain vaccination) among HCWs decreased to 27.3% (as shown in Figure 1). Additionally, ~12% of those who had already received the first dose were uncertain about or not willing to accept the second dose.

### Demographic Characteristics, Socio-Economic Factors, and Health-Related Factors

The description regarding demographic characteristics, socio-economic factors, and health-related factors for the total sample and by time periods were similar (Table 1). Overall, 81.6% were female, the mean age was 42.6 years (interquartile range: IQR = 33–52), and the majority (56.4%) were married. The majority of the respondents were from the northeastern (34.8%) and central (32.9%) regions. In total, 31.9% were nurses and 18.9% were of village health volunteers and migrant health volunteers. The respondents mainly worked in primary care units and community hospitals, including secondary and tertiary hospitals. The majority had received influenza vaccines (85.0%) and did not have COVID-19 (98.3%).

**Table 1:** Demographic characteristics, socio-economic factors, and health-related factors of the study population and by time period.

**Characteristics**	**Total (*N* = 82,387)**	**Pre-vaccine arrival period (*N* = 55,068)**	**Post-vaccine arrival period (*N* = 27,319)**
**Age (mean** **±SD) (IQR)**	42.6 ± 11.7 (33–52)	41.9 ± 11.7 (32–51)	44.1 ± 11.6 (34–53)
**Gender**
Female	67,261 (81.6%)	44,942 (81.6%)	22,319 (81.7%)
**Marital status**
Single	28,993 (35.2%)	20,229 (36.7%)	8,764 (32.1%)
Marriage	46,478 (56.4%)	30,457 (55.3%)	16,021 (58.6%)
Separated, widowed or divorced	6,916 (8.4%)	4,382 (8.0%)	2,534 (9.3%)
**Religion**
Buddhist	78,966 (95.8%)	52,841 (96.0%)	26,125 (95.6%)
**Work region**			
Bangkok	15,688 (19.0%)	12,365 (22.5%)	3,323 (12.2%)
Central	21,117 (32.9%)	15,946 (29.0%)	11,171 (40.9%)
North	4,731 (5.7%)	3,374 (6.0%)	1,357 (5.0%)
Northeast	28,658 (34.8%)	20,265 (36.8%)	8,393 (30.7%)
South	6,193 (7.6%)	3,118 (5.7%)	3,075 (11.2%)
**Work location**
Rural	43,405 (52.7%)	27,987 (50.8%)	15,418 (56.4%)
**Frontline COVID-19 worker**
Yes	55,874 (67.8%)	36,832 (66.9%)	19,042 (69.7%)
**Occupation**
Doctors	7,400 (9.0%)	4,492 (8.2%)	2,908 (10.6%)
Dentists	2,107 (2.6%)	1,061 (1.9%)	1,046 (3.8%)
Pharmacist	2,606 (3.2%)	1,722 (3.1%)	884 (3.2%)
Nurses	26,300 (31.9%)	18,394 (33.4%)	7,906 (28.9%)
Medical laboratories	2,840 (3.4%)	1,872 (3.4%)	968 (3.5%)
Patient aids	7,274 (8.8%)	5,484 (10.0%)	1,790 (6.6%)
Village health volunteers and migrant health volunteers	15,593 (18.9%)	9,172 (16.7%)	6,421 (23.5%)
Public health officers	7,787 (9.5%)	4,956 (9.0%)	2,831 (10.4%)
Others	10,480 (12.7%)	7,915 (14.3%)	2,565 (9.5%)
**Type of workplace**
Primary care unit and community hospital	35,792 (43.4%)	23,209 (42.1%)	12,583 (46.1%)
Secondary and tertiary hospital	20,669 (25.1%)	13,542 (24.6%)	7,127 (26.1%)
Specialized hospitals of government departments and university hospital	11,571 (14.0%)	9,547 (17.3%)	2,024 (7.4%)
General government units and supporting unit	6,059 (7.4%)	3,616 (6.6%)	2,443 (8.9%)
Private unit and other office	8,296 (10.1%)	5,154 (9.4%)	3,142 (11.5%)
**Have more than one workplace**
Yes	14,463 (17.6%)	9,188 (16.7%)	5,275 (19.3%)
**Have health condition**
Yes	13,637 (16.6%)	8,983 (16.3%)	4,654 (17.0%)
**Had influenza vaccines before**
Yes	70,085 (85.0%)	47,415 (86.1%)	22,670 (83.0%)
No	10,101 (12.3%)	6,182 (11.2%)	3,919 (14.3%)
Uncertain	2,201 (2.7%)	1,471 (2.7%)	730 (2.7%)
**Had history of COVID-19 infection**
No	80,952 (98.3%)	54,133 (98.3%)	26,819 (98.2%)
Yes	1,435 (1.7%)	935 (1.7%)	500 (1.8%)

*SD, standard deviation; IQR, Interquartile range*.

The descriptive findings of respondents' intention to receive the COVID-19 vaccine by period are shown in Supplementary Material 2. HCWs who were willing to accept the COVID-19 vaccine appeared to be older in the post-vaccine arrival period (pre-vaccine arrival period: 47.1 ± 11.9 for those who were willing to accept the vaccines vs. 41.9 ± 11.7 for those who said no or were uncertain; post-vaccine arrival period with 0 dose: 46.6 ± 12.8 vs. 40.8 ± 12.7, and with 1 dose: 41.9 ± 12.6 vs. 36.7 ± 12.9).

### Perceived Risk for COVID-19

Respondents' perceived risk of the COVID-19 infection was studied and is reported in Table 2. Most participants expressed concerns about exposure to the infection from workplace (55.7%) more than from their homes and community (44.3%). Approximately 40% reported neither living with older adults nor people with chronic diseases (chronic patients) in both the pre-vaccine and post-vaccine arrival periods. Furthermore, around two-thirds of HCWs perceived a moderate risk of infection, regardless of actual vaccine availability.

**Table 2:** Perceived risk of COVID-19 by health care workers.

**Characteristics**	**Total (*N* = 82,387)**	**Pre-vaccine arrival period (*N* = 55,068)**	**Post-vaccine arrival period (*N* = 27,319)**
**Had direct contact in the screening and/or caring of COVID-19**	55,874 (67.8%)	36,832 (66.9%)	19,042 (69.7%)
**patients (Frontline workers)**			
**Perceived risk of COVID-19**
No risk	848 (1.0%)	507 (0.9%)	341 (1.2%)
Low risk	4,621 (5.6%)	3,322 (6.0%)	1,299 (4.8%)
Moderately low risk	13,417 (16.3%)	9,528 (17.3%)	3,889 (14.2%)
Moderate risk	28,795 (35.0%)	19,636 (35.7%)	9,159 (33.5%)
Moderately high risk	24,820 (30.1%)	15,886 (28.8%)	8,934 (32.7%)
High risk	9,886 (12.0%)	6,189 (11.2%)	3,697 (13.5%)
**Living conditions as risk of COVID-19**
Living with older adults (≥60 years)	27,854 (33.8%)	18,338 (33.3%)	9,516 (34.8%)
Living with chronic patients	6,962 (8.5%)	4,464 (8.1%)	2,498 (9.1%)
Living with both older adults and chronic patients	11,958 (14.5%)	7,758 (14.1%)	4,200 (15.4%)
Living with neither older adults nor chronic patients	35,613 (43.2%)	24,508 (44.5%)	11,105 (40.6%)
**Location perceived to be highest risk of COVID-19 infection**
Home and community	36,491 (44.3%)	24,586 (44.6%)	11,905 (43.6%)
Workplace	45,896 (55.7%)	30,482 (55.4%)	15,414 (56.5%)

### Perceived Enablers and Barriers of COVID-19 Vaccination

The list of top three enablers and barriers selected by HCWs are presented in Table 3, before and after the availability of COVID-19 vaccines in Thailand. The enablers and barriers to the receipt of COVID-19 vaccinations were similar in both periods. The top enablers for HCWs were if WHO or FDA in Thailand recommended the vaccine, and their belief on whether the vaccine would stop infections. The topmost barrier was the concern about the efficacy of COVID-19 vaccines.

**Table 3:** Top 3 enablers and barriers for COVID-19 vaccination before and after COVID-19 vaccination.

**Factors**	**Pre-vaccine arrival period**	**Post-vaccine arrival period**
Enabler	1. If WHO or Thai FDA recommended COVID-19 vaccination 2. Believe that the COVID-19 vaccine will stop infection 3. Believe that the COVID-19 vaccine will prevent transmission	1. If WHO or Thai FDA recommended COVID-19 vaccination 2. Believe that the COVID-19 vaccine will prevent mortality 3. Believe that the COVID-19 vaccine will prevent transmission
Barriers	1. Concern about the efficacy of COVID-19 vaccination 2. Concern about the short-term side effects of COVID-19 vaccination 3. Concern about the long-term side effects of COVID-19 vaccination	1. Concern about the efficacy of COVID-19 vaccination 2. Concern about the long-term side effects of COVID-19 vaccination 3. Concern about the short-term side effects of COVID-19 vaccination

### Factors Associated With COVID-19 Vaccine Hesitancy

This section reports the findings on factors associated with COVID-19 vaccine hesitancy by HCWs over time from the pre-vaccine arrival period to post-vaccine arrival period. Results from multinomial logistic regression analysis are presented in Table 4. Specifically, this study examined factors associated with vaccine hesitancy (with the outcome being yes, no, or uncertain) among participants who had not received the vaccination in the pre-vaccine arrival period (*N* = 55,068) and post-vaccine arrival period (*N* = 11,066). Those who received two doses were excluded.

**Table 4:** Factors associated with COVID-19 vaccine hesitancy among participants who have not received vaccination between in the pre- and post-vaccine period.

**Variables**	**Multinomial logistic regression (Relative Risk Ratio: RRR)**			
	**Pre-vaccine arrival period (*****N*** **=** **55,068)**		**Post-vaccine arrival period (*****N*** **=** **11,066)**	
	**RRR for those who declined COVID-19 vaccines (compared to those who will receive the vaccine)**	**RRR for those who are uncertain about COVID-19 vaccines (compared to those who will receive the vaccine)**	**RRR for those who declined COVID-19 vaccines (compared to those who will receive the vaccine)**	**RRR for those who are uncertain about COVID-19 vaccines (compared to those who will receive the vaccine)**
**Age**	1.01 (1.00–1.01)***	1.00 (0.99–1.00)*	0.99 (0.98–1.00)***	0.99 (0.99–1.00)***
**Gender (Ref: Male)**				
Female	1.09 (0.99–1.20)*	1.26 (1.18–1.34)***	1.20 (1.00–1.44)**	1.24 (1.09–1.41)***
**Marital status (Ref: Single)**				
Marriage	0.89 (0.82–0.97)***	0.96 (0.91–1.01)	1.01 (0.85–1.20)	0.93 (0.82–1.05)
Separated, widowed or divorced	0.93 (0.80–1.08)	0.99 (0.90–1.09)	1.32 (1.02–1.69)**	0.94 (0.78–1.12)
**Religion (Ref: Others)**				
Buddhist	0.67 (0.57–0.79)***	0.81 (0.72–0.91)***	0.77 (0.57–1.04)*	0.79 (0.64–0.98)*
**Work region (Ref: Bangkok)**				
Central	0.86 (0.78–0.95)***	0.92 (0.86–0.99)**	1.24 (0.90–1.70)	1.18 (0.95–1.47)
North	0.97 (0.83–1.13)	0.97 (0.88–1.08)	0.91 (0.59–1.39)	1.27 (0.95–1.68)
Northeast	1.01 (0.90–1.14)***	0.89 (0.83–0.96)***	1.09 (0.79–1.51)	1.23 (0.98–1.54)*
South	0.70 (0.58–0.84)***	0.98 (0.88–1.09)	0.80 (0.57–1.14)	0.85 (0.67–1.07)
**Work location (Ref: Urban)**				
Rural	1.10 (1.00–1.21)**	0.99 (0.93–1.05)	1.11 (0.96–1.29)	1.06 (0.96–1.17)
**Frontline COVID-19 worker (Ref: Yes)**				
No	1.11 (1.03–1.20)***	1.15 (1.09–1.21)***	0.91 (0.77–1.07)	0.94 (0.84–1.05)
**Occupation (Ref: Public health officers)**				
Doctors	0.99 (0.83–1.17)	0.95 (0.85–1.07)	0.70 (0.47–1.03)*	0.62 (0.47–0.82)***
Dentists	0.84 (0.65–1.08)	0.96 (0.80–1.15)	0.42 (0.25–0.69)***	0.71 (0.52–0.98)**
Pharmacists	1.12 (0.91–1.37)	1.00 (0.86–1.16)	0.57 (0.35–0.93)**	0.63 (0.46–0.87)***
Nurses	1.07 (0.93–1.22)	1.03 (0.94–1.13)	1.09 (0.81–1.46)	1.04 (0.84–1.28)
Medical laboratories	0.79 (0.64–0.97)**	0.87 (0.75–1.00)**	1.08 (0.69–1.69)	0.88 (0.62–1.24)
Patient aids	0.62 (0.52–0.73)***	0.81 (0.72–0.90)***	1.25 (0.82–1.89)	0.90 (0.65–1.23)
Village health volunteers and migrant health volunteers	0.31 (0.26–0.37)***	0.84 (0.76–0.93)***	1.72 (1.29–2.29)***	1.80 (1.48–2.19)***
Others	0.68 (0.59–0.79)***	0.86 (0.78–0.95)***	0.66 (0.47–0.93)*	0.61 (0.48–0.78)*
**Type of workplace (Ref: Primary care unit and community hospital)**				
Secondary and tertiary hospital	0.83 (0.75–0.92)***	0.90 (0.84–0.96)***	0.85 (0.69–1.06)	0.86 (0.73–1.01)*
Specialized hospital of government departments and university hospital	1.00 (0.88–1.13)	0.99 (0.92–1.08)	0.86 (0.63–1.18)	0.96 (0.76–1.20)
General government units and supporting unit	1.12 (0.96–1.30)	1.05 (0.96–1.16)	0.85 (0.67–1.08)	0.98 (0.84–1.14)
Private unit and other office	0.73 (0.63–0.84)***	0.90 (0.83–0.99)**	0.53 (0.40–0.70)***	0.67 (0.56–0.79)***
**Have more than one workplace (Ref: Yes)**				
No	1.01 (0.92–1.12)	1.09 (1.02–1.16)***	1.30 (1.10–1.54)***	1.01 (0.90–1.13)
**Have health condition (Ref: Yes)**				
No	0.93 (0.85–1.03)	0.96 (0.91–1.02)	0.77 (0.66–0.91)***	0.82 (0.73–0.92)***
**Had influenza vaccines before (Ref: Yes)**				
No	2.69 (2.42–2.98)***	1.35 (1.26–1.45)***	1.56 (1.34–1.81)***	1.16 (1.04–1.29)***
Uncertain	1.01 (0.78–1.30)	1.29 (1.12–1.49)***	1.02 (0.73–1.43)	1.37 (1.09–1.72)***
**Had COVID-19 (Ref: Yes)**				
No	0.74 (0.38–1.43)	1.40 (0.87–2.26)	0.83 (0.47–1.47)	1.29 (0.86–1.95)
**Would recommend others to receive COVID−19 vaccine (Ref: Yes)**				
No	103.56 (89.02–120.48)**	10.03 (8.62–11.66)***	56.43 (40.29–79.03)***	5.51 (3.87–7.84)***
Uncertain	17.73 (16.36–19.22)***	14.73 (14.05–15.44)***	10.81 (9.28–12.61)***	9.10 (8.06–10.26)***
**Constant**	0.07 (0.03–0.13)***	0.14 (0.09–0.24)***	0.17 (0.10–0.31)***	**0.71 (0.47–1.07)**
**Log likelihood**	**−38,829.39**		**−9,469.2668**	
**Prob** **>** **chi**^**2**^	**0.0000**		**0.0000**	
***Pseudo R* ^2^**	**0.2345**		**0.1483**	

*p ≤ 0.1, **p ≤ 0.05, ***p ≤ 0.01; results from multinomial logistic regression models.

In the pre-vaccine arrival period, HCWs likely to decline a COVID-19 vaccine, compared to those willing to accept a COVID-19 vaccine, were generally older (RRR: 1.01, 95% CI: 1.00–1.01), were mostly single (RRR: 0.89, 95% CI: 0.82–0.97), were mainly Buddhist (RRR: 0.67, 95% CI: 0.57–0.79), mainly worked in rural areas (RRR: 1.10, 1.00–1.21), were generally not frontline COVID-19 workers (RRR: 1.11, 95% CI: 1.03–1.20), had not received Influenza vaccines before (RRR: 2.69, 95% CI: 2.42–2.98), and would not recommend that others receive a COVID-19 vaccine (RRR: 103.56, 95% CI: 89.02–120.48). Across different working regions, HCWs working in the northeastern region were most likely to decline COVID-19 vaccines, and HCWs working in the southern region were least likely to decline COVID-19 vaccines. HCWs working in medical laboratories (RRR: 0.79, 95% CI: 0.64–0.97), working as patient aids (RRR: 0.62, 95% CI: 0.52–0.73), and village health volunteers and migrant health volunteers (RRR: 0.31, 95% CI: 0.26–0.37) were less likely to decline COVID-19 vaccines compared to public health officers. HCWs working at secondary and tertiary hospitals (RRR: 0.83, 95% CI: 0.75–0.92), private units, and other offices (RRR: 0.73, 95% CI: 0.63–0.84) were less likely to decline COVID-19 vaccines compared to HCWs working at primary care unit and community hospital.

When comparing HCWs who were uncertain about COVID-19 vaccines with those who stated their preference for COVID-19 vaccines, the abovementioned factors had similar impacts (with different magnitudes). The exceptions included age, work region, number of workplaces, and influenza vaccine status. Younger HCWs (RRR: 1.00, 95% CI: 0.99–1.00) were more likely to be uncertain, although the result was not significant at 5%. HCWs working in Bangkok, the northern region, and southern region were more likely to be uncertain compared to HCWs working in the central region and northeastern region. HCWs having only one workplace (RRR: 1.09, 95% CI: 1.02–1.16) were more likely to be uncertain compared to HCWs having more than one workplace. HCWs who were uncertain about whether they had received the influenza vaccine before (RRR: 1.29, 95% CI: 1.12–1.49) were also more likely to be uncertain about whether to receive the COVID-19 vaccine.

In the post-vaccine arrival period, HCWs were more likely to decline COVID-19 vaccines if they were younger (RRR: 0.99, 95% CI: 0.98–1.00), were female (RRR: 1.20, 95% CI: 1.00–1.44), were separated or widowed, or divorced (RRR: 1.32, 95% CI: 1.02–1.69), worked in primary care units and community hospitals (RRR: 0.53, 95% CI: 0.40–0.70), had only one workplace (RRR: 1.30, 95% CI: 1.10–1.54), had health conditions (RRR: 0.77, 95% CI: 0.66–1.54), had not received influenza vaccines before (RRR: 1.56, 95% CI: 1.34–1.81), were uncertain about recommending that others receive a COVID-19 vaccine (RRR: 56.43, 95% CI: 40.29–79.03), and were not willing to make such a recommendation (RRR: 10.81, 95% CI: 9.28–12.61). In terms of occupation, HCWs who worked as village health volunteers and migrant health volunteers (RRR: 1.72, 95% CI: 1.29–2.29) were more likely to decline COVID-19 vaccines than public health officers. Pharmacists (RRR: 0.57, 95% CI: 0.35–0.93) and dentists (RRR: 0.42, 95% CI: 0.25–0.69) were less likely to decline COVID-19 vaccines than public health officers. A similar pattern can be observed in the results when comparing HCWs that were uncertain about COVID-19 vaccines to those who stated their preference for them. The exceptions included marital status, number of workplaces, and influenza vaccine status. Marital status and number of workplaces had no impact on the likelihood of being uncertain about COVID-19 vaccines. Doctors (RRR: 0.62, 95% CI: 0.47–0.82) were less likely to be uncertain about receiving COVID-19 vaccines compared to public health officers. HCWs who were uncertain about whether they received an influenza vaccine before (RRR: 9.10, 95% CI: 8.06–10.26) were also more likely to be uncertain about receiving COVID-19 vaccines.

When comparing between the pre- and post-vaccine arrival periods, older HCWs were more likely to decline COVID-19 vaccines in the pre-vaccine arrival period; on the other hand, older HCWs became less likely to decline and be uncertain about receiving a COVID-19 vaccine in the post-vaccine arrival period. Compared to HCWs in the urban region, HCWs in the rural region were more likely to decline a vaccine in the pre-vaccine arrival period (RRR: 1.10, 95% CI: 1.00–1.21), but there was no difference in this trend in the post-vaccine arrival period. HCWs who were not frontline workers were more likely to decline or be uncertain about receiving the vaccines in the pre-vaccine arrival period, but not in the post-vaccine arrival period. Additionally, respondents in certain occupations changed their perception regarding receiving COVID-19 vaccines in the post-vaccine arrival period; for instance, medical laboratories, patient aids, and village health volunteers were more likely to decline or be uncertain about receiving COVID-19 vaccines than public health officers in both periods. Similarly, HCWs working in secondary and tertiary hospitals also changed their vaccine hesitancy from having them to none in the post-vaccine arrival period.

## Discussion

This study examined factors affecting COVID-19 vaccine hesitancy among health care workers in Thailand before and after vaccines became available by using primary data collected from an online survey. The reasons for people choosing not to be vaccinated are complex; for example, a vaccines advisory group to WHO identified complacency, inconvenience in accessing vaccines, and lack of confidence as key reasons underlying hesitancy (18). Given the important role of HCWs in the community regarding their roles in caring for patients and in educating people about health-related matters, they remain the most trusted advisors and influencers of vaccination decisions and they should be supported so they can provide trusted and credible information on vaccines to the general population. Given the current evidence on vaccine effectiveness against infection and preventing serious illness, it is important to have a high vaccine coverage of COVID-19 vaccinations among HCWs to prevent the shortage of HCWs during the time of public health crisis (19), and to increase the public confidence in the COVID-19 vaccination policy of the country (9). If HCWs are not hesitant to receive COVID-19 vaccines, they may be more likely to recommend and encourage other eligible people to be vaccinated, which was confirmed by this study's findings.

As stated before, it was found that in the pre-vaccine arrival period, ~55% of HCWs were willing to accept the vaccine, 35% were uncertain, and 10% declined. In the post-vaccine arrival period, the vaccine hesitancy among HCWs reduced to 27%. This study reported a higher rate of vaccine hesitancy than those reported in existing literature (11, 20–24) especially in the pre-vaccine arrival period. After the vaccines became available, there was a reduction in vaccine hesitancy, which may be attributed to the fact that the survey period covered the time when COVID-19 cases spiked in Thailand.

Socio-demographic factors, such as being female, younger age, living in Bangkok, working as medical laboratory workers, patient aids, village health volunteers, and migrant health volunteers, not receiving the influenza vaccine before, and the preference not to recommend that others receive COVID-19 vaccine were found to be the significant factors explaining vaccination hesitancy. These findings supported existing literature in that they confirmed that males are more likely to take a chance and try new options (in this case, COVID-19 vaccine) and those with a history of seasonal influenza vaccination were more open to accepting COVID-19 vaccines (11, 21, 23, 25). These findings led to a better plan for target communications such as using social media to promote COVID-19 vaccine uptake among young female healthcare workers, and providing vaccine information through opinion leaders in hospitals and professional societies. These initiatives aim to improve the uptake of COVID-19 vaccines in clinical settings. Recently, the study findings were also used as one of inputs for designing COVID-19 booster vaccination among healthcare workers.

Our findings in the post-vaccine arrival period showed additional factors influencing vaccine hesitancy, including working in a rural area and having health conditions. These findings highlighted that vaccine recommendations and vaccine confidence can boost vaccine acceptance. In contrast, concerns regarding vaccine efficacy and safety were barriers to accepting the vaccine (11, 26–29). It is to be noted that similar studies about vaccine acceptance or hesitancy among HCWs were recently conducted in other countries. Those studies emphasized that female health care workers were less likely to accept COVID-19 vaccines than males. One of the main barriers to vaccination acceptance was found to be the mistrust of the vaccine (30).

In addition, married HCWs were less likely to decline or be uncertain to receive COVID-19 vaccines in both periods, possibly due to concerns for the safety of their families. Geographical areas appeared to be a factor in vaccine hesitancy; as highlighted by existing literature and this study's findings in the pre-vaccine arrival period, this threat is more common in rural areas (31). After the arrival of the vaccines, the effects of geographical areas diminished. Comparing both the periods, vaccine acceptance changed for frontline HCWs and for people in certain occupations (11), who were all more hesitant about receiving a COVID-19 vaccine before its arrival but not after. Potential explanations could include the peer effect (e.g., seeing their colleagues getting vaccinated and remaining healthy), feeling more confident with the presence of the vaccine, and the public campaigns in promoting COVID-19 vaccines during that time.

The findings can be used to support the management of COVID-19 vaccinations for HCWs in the future for booster dose(s) and in other settings for future outbreaks. First, it is important to monitor real-world vaccine safety and effectiveness and share information with HCWs, given that concerns over this information were top barriers to vaccine acceptance. Vaccine hesitancy issue among HCWs is an alarming sign suggesting that HCWs perceived the effects of the COVID-19 vaccination as a higher risk to their lives than contracting the COVID-19 virus itself. Having a tailored program for those who declined or remained uncertain about getting vaccinated could minimize this hesitancy and subsequently influence the potential success of vaccination programs. For example, female HCWs living in the rural areas and being in certain occupations (e.g., nurses) should receive tailored information to support their vaccination plan. Second, another study has shown that a large number of HCWs in Thailand also contracted the COVID-19 virus from their peers (through having lunch and dinner together, for instance) (32). Therefore, there may be a misunderstanding that HCWs with adequate Personal Protective Equipment or working with non-COVID patients are safe. Information needs to be communicated to them to ensure that they are not underestimating the infection risk.

### Strengths and Limitations of the Study

The strength of our study was that the information on COVID-19 vaccine hesitancy was examined in both the pre-vaccine arrival and the post-vaccine arrival periods in Thailand, which can shed light on the behavioral changes which took place after the arrival of vaccines. An additional strength of this study lies in its sample size, which was substantial compared to previous studies. In this regard, this level of response was possible with the support from the Thai MOPH along with several communication strategies to reach the study population. The information from this analysis may also be applicable to other settings with a similar context and can help support the design of programs/interventions to minimize vaccine hesitancy. Generalisability of the results may depend on many factors, for example, the level of local transmission and prior vaccine acceptance among HCWs. The first survey was conducted in early 2021 when the local epidemic was relatively low. While this study's results may not be applicable to settings with different levels of local transmission and vaccine acceptance among HCWs, the survey tools are transferable (usable in) to these settings. Nevertheless, there are limitations to consider when interpreting the findings. This study was cross-sectional at two different time points. The surveys were not linked; thus, the pattern or trend could not be assessed. The differences in results during both the periods could be due to different sample pools and sample sizes. However, the chance of this issue was minimized as similar methods and channels for the recruitment of participants were used for the two time points. Future research could consider a method where same participants were followed over time. Additionally, there may be unknown confounders which were not included due to the lack of data, as noticeable in the low *R*-squared value; however, this study attempted to collect data on relevant factors influencing vaccine hesitancy based on our initial literature review.

## Conclusion

This study's findings describe how policies can be addressed to reduce vaccine hesitancy. This study also highlights HCWs' characteristics (including gender, work region, and occupation) and the reasons they cited for their vaccine acceptance or hesitance. These findings are critical given Thailand's efforts to fight the COVID-19 pandemic with various public health interventions, including COVID-19 vaccines, and may be helpful to support other settings in their fight against this pandemic.

## Data Availability Statement

The original contributions presented in the study are included in the article/Supplementary Material, further inquiries can be directed to the corresponding author/s.

## Ethics Statement

Ethical approval was obtained from the Institute for the Development of Human Research Protections (IHRP) in Thailand. Participation was voluntary and only non-identifiable data were collected. The patients/participants provided their written informed consent to participate in this study.

## Author Contributions

CP, YW, and WI analyzed and interpreted data. All authors designed the study, including objectives, questionnaire, data collection, and approved the final draft.

## Funding

This study was funded by the Health Systems Research Institute (https://hsri.or.th/researcher), grant number 64134002RM011L0. The funder of the study had no role in study design, data collection, data analysis, data interpretation, or writing of the report. The Health Intervention and Technology Assessment Program (HITAP) is a semi-autonomous research unit in the Ministry of Public Health, Thailand, supports evidence-informed priority-setting and decision-making for healthcare. HITAP was funded by National and International Public Funding Agencies. HITAP's international work was supported by the International Decision Support Initiative (iDSI), with the aim of providing technical assistance on health intervention and technology assessment to governments in low-income and middle-income countries. iDSI was funded by the Bill & Melinda Gates Foundation (OPP1202541), the UK's Department for International Development, and the Rockefeller Foundation.

## Author Disclaimer

The findings, interpretations and conclusions expressed in this article do not necessarily reflect the views of the funding agencies.

## Conflict of Interest

The authors declare that the research was conducted in the absence of any commercial or financial relationships that could be construed as a potential conflict of interest.

## Publisher's Note

All claims expressed in this article are solely those of the authors and do not necessarily represent those of their affiliated organizations, or those of the publisher, the editors and the reviewers. Any product that may be evaluated in this article, or claim that may be made by its manufacturer, is not guaranteed or endorsed by the publisher.
