# Supplementary material for: COVID-19 Vaccine Hesitancy Among Health Care Workers in Thailand: The Comparative Results of Two Cross-Sectional Online Surveys Before and After Vaccine Availability
Source: Front Public Health. 2022 Aug 1;10:834545. doi: 10.3389/fpubh.2022.834545 (PMC9376379; doi:10.3389/fpubh.2022.834545)
Supplement: Supplementary file 1 [file Table_1.docx]

**Supplementary 2: Demographic characteristics, socio-economic factors, and health-related factors of the study population and by vaccination status and time period**

| **Characteristics** | **Pre-vaccine arrival period (N = 55,068)** | | | **Post-vaccine arrival period**  **(N = 27,319)** | | | | | | |
| --- | --- | --- | --- | --- | --- | --- | --- | --- | --- | --- |
|  | **0 dose** | | | **0 doses** | | | **1 dose** | | | **2 doses** |
|  | Yes 55% (30,300) | No  10% (5,277) | Uncertain  35% (19,491) | Yes  45%  (4,945) | No  14%  (1,594) | Uncertain  41%  (4,527) | Yes  89%  (10,487) | No  3%  (302) | Uncertain  9%  (1,035) | Yes  27%  (4,429) |
| **Age (mean ± SD)** | 43 ± 12 | 40 ± 12 | 40 ± 12 | 47 ± 12 | 45 ± 13 | 45 ± 12 | 43 ± 11 | 37 ± 12 | 36 ± 11 | 36 ± 11 |
| **Gender** | | | | | | | | | | |
| Female | 79% (23,853) | 83%  (4,381) | 86%  (16,708) | 81%  (3,981) | 85%  (1,350) | 86%  (3,894) | 79%  (8,245) | 86%  (259) | 83%  (859) | 78%  (3,468) |
| **Marital status** | | | | | | | | | | |
| Single | 32%  (9,829) | 45%  (2,381) | 41%  (8,019) | 25%  (1,229) | 26%  (422) | 25%  (1,144) | 34%  (3,616) | 53%  (160) | 52%  (534) | 37%  (1,659) |
| Marriage | 59%  (17,860) | 48%  (2,538) | 52%  (10,059) | 65%  (3,203) | 61%  (972) | 64%  (2,889) | 57%  (5,965) | 38%  (114) | 43%  (449) | 55%  (2,429) |
| Separated, widowed or divorced | 9%  (2,611) | 358  (7%) | 7%  (1,413) | 10%  (513) | 13%  (200) | 11%  (494) | 9%  (906) | 9%  (28) | 5%  (52) | 8%  (341) |
| **Religion** | | | | | | | | | | |
| Buddhist | 96%  (29,234) | 95%  (5,004) | 95%  (18,603) | 95%  (4,720) | 95%  (1,511) | 95%  (4,313) | 96%  (10,098) | 90%  (273) | 93%  (965) | 96%  (4,245) |
| **Work region** | | | | | | | | | | |
| Bangkok | 19%  (5,645) | 28%  (1,491) | 27%  (5,229) | 11%  (524) | 6%  (98) | 6%  (273) | 12%  (1,299) | 8%  (23) | 11%  (116) | 22%  (990) |
| Central | 29%  (8,677) | 29%  (1,560) | 30%  (5,759) | 39%  (1,942) | 46%  (729) | 41%  (1,837) | 39%  (4,126) | 34%  (103) | 32%  (326) | 47%  (2,072) |
| North | 6%  (1,769) | 7%  (353) | 6%  (1,252) | 5%  (260) | 4%  (67) | 5%  (227) | 5%  (502) | 3%  (10) | 5%  (55) | 5%  (236) |
| Northeast | 41%  (12,479) | 32%  (1,696) | 31%  (6,090) | 29%  (1,439) | 32%  (508) | 35%  (1,570) | 34%  (3,603) | 47%  (143) | 45%  (461) | 15%  (669) |
| South | 6%  (1,730) | 4%  (227) | 6%  (1,161) | 16%  (780) | 12%  (192) | 13% (584) | 9%  (957) | 8%  (23) | 7%  (77) | 10%  (426) |
| **Work location** | | | | | | | | | | |
| Rural | 29%  (8,787) | 20%  (1,076) | 21%  (4,125) | 50%  (2,488) | 52%  (836) | 50%  (2,277) | 62%  (6,454) | 62%  (186) | 60%  (626) | 58%  (2,551) |
| **Frontline COVID-19 worker** | | | | | | | | | | |
| No | 29%  (8,648) | 40%  (2,085) | 38%  (7,503) | 35%  (1,708) | 28%  (439) | 28%  (1,247) | 33%  (3,417) | 19%  (56) | 28%  (289) | 25%  (1,121) |
| **Occupation** | | | | | | | | | | |
| Doctors | 8%  (2,555) | 9%  (484) | 7%  (1,453) | 8%  (383) | 4%  (65) | 4%  (172) | 10%  (1,072) | 19%  (57) | 13%  (138) | 23%  (1,021) |
| Dentists | 2% (526) | 2%  (116) | 2%  (419) | 4%  (207) | 2%  (25) | 3%  (130) | 4%  (403) | 6%  (19) | 5%  (53) | 5%  (209) |
| Pharmacist | 3%  (836) | 4%  (228) | 3%  (658) | 5%  (240) | 2%  (36) | 3%  (116) | 3%  (350) | 3%  (8) | 4%  (40) | 2%  (94%) |
| Nurses | 28%  (8,611) | 44%  (2,301) | 38%  (7,482) | 19%  (917) | 22%  (347) | 19%  (867) | 35%  (3,641) | 38%  (114) | 42%  (439) | 36%  (1,581) |
| Medical laboratories | 3%  (987) | 4%  (207) | 3%  (678) | 3%  (137) | 3%  (47) | 2%  (109) | 4%  (417) | 3%  (8) | 4%  (46) | 5%  (204) |
| Patient aids | 10%  (3,166) | 8%  (425) | 10%  (1,893) | 3%  (152) | 5%  (76) | 3%  (137) | 10%  (1,081) | 5%  (14) | 8%  (86) | 6%  (244) |
| Village health volunteers and migrant Health Volunteers | 22%  (6,540) | 6%  (320) | 12%  (2,312) | 40%  (1,995) | 49%  (782) | 53%  (2,405) | 7%  (766) | 15%  (45) | 7%  (71) | 8%  (357) |
| Public health officers | 10%  (2,957) | 9%  (465) | 8%  (1,534) | 8%  (399) | 6%  (103) | 7%  (306) | 14%  (1,517) | 8%  (23) | 8%  (78) | 9%  (405) |
| Others | 14%  (4,122) | 14%  (731) | 16%  (3,062) | 10%  (515) | 7%  (113) | 6%  (285) | 12%  (1,240) | 5%  (14) | 8%  (84) | 7%  (314) |
| **Type of workplace** | | | | | | | | | | |
| Primary care unit and community hospital | 47%  (14,253) | 34%  (1,819) | 37%  (7,137) | 51%  (2,515) | 60%  (961) | 61%  (2,770) | 42%  (4,445) | 44%  (133) | 43%  (448) | 30%  (1,311) |
| Secondary and tertiary hospital | 23%  (6,835) | 27%  (1,442) | 27%  (5,265) | 15%  (747) | 17%  (271) | 14%  (630) | 32%  (3,350) | 34%  (102) | 35%  (367) | 37%  (1,660) |
| Specialized hospitals of government departments and university hospital | 14%  (4,264) | 23%  (1,198) | 21%  (4,085) | 6%  (286) | 5%  (87) | 5%  (236) | 7%  (722) | 10%  (30) | 8%  (86) | 13%  (577) |
| General government units and supporting unit | 7%  (2,105) | 7%  (345) | 6%  (1,166) | 11%  (529) | 9%  (141) | 10%  (461) | 8%  (866) | 5%  (15) | 4%  (43) | 9%  (388) |
| Private unit and other office | 9%  (2,843) | 9%  (473) | 9%  (1,838) | 18%  (868) | 8%  (134) | 10%  (430) | 11%  (1,104) | 7%  (22) | 9%  (91) | 11%  (493) |
| **Have more than one workplace** | | | | | | | | | | |
| No | 82%  (24,906) | 84%  (4,455) | 85%  (16,519) | 77%  (3,832) | 84%  (1,334) | 79%  (3,567) | 84%  (8,817) | 80%  (242) | 84%  (871) | 76%  (3,381) |
| **Have health condition** | | | | | | | | | | |
| No | 83%  (25,116) | 85%  (4,483) | 85%  (16,486) | 81%  (4,025) | 81%  (1,286) | 80%  (3,633) | 84%  (8,826) | 88%  (266) | 86%  (889) | 84%  (3,740) |
| **Had influenza vaccines before** | | | | | | | | | | |
| Yes | 87%  (26,279) | 81%  (4,291) | 86%  (16,845) | 75%  (3,726) | 67%  (1,065) | 70%  (3,187) | 90%  (9,428) | 88%  (267) | 89%  (919) | 92%  (4,078) |
| No | 11%  (3,213) | 17%  (899) | 11%  (2,070) | 21%  (1,054) | 29%  (470) | 24%  (1,089) | 8%  (876) | 11%  (32) | 8%  (83) | 7%  (315) |
| Uncertain | 2%  (808) | 2%  (87) | 3%  (576) | 3%  (165) | 4%  (59) | 6%  (251) | 2%  (183) | 1%  (3) | 3%  (33) | 1%  (36) |
| **Had COVID-19** | | | | | | | | | | |
| No | 99%  (30,300) | 99%  (5,263) | 99%  (19,461) | 99%  (4,933) | 99%  (1,591) | 99%  (4,514) | 99%  (10,407) | 98%  (298) | 99%  (1,025) | 99%  (4,399) |
