# Supplementary material for: COVID-19 Vaccine Hesitancy Among Health Care Workers in Thailand: The Comparative Results of Two Cross-Sectional Online Surveys Before and After Vaccine Availability
Source: Front Public Health. 2022 Aug 1;10:834545. doi: 10.3389/fpubh.2022.834545 (PMC9376379; doi:10.3389/fpubh.2022.834545)
Supplement: Supplementary file 2 [file Data_Sheet_1.docx]

# Supplementary Material: Questionnaire used in the study

Health Intervention and Technology Assessment Program Thailand (HITAP) gently request your cooperation to complete this survey. Your responses will support us in addressing any issues regarding the COVID-19 vaccine acceptance in Thailand, and all responses will be kept confidential. You will take approximately five minutes to complete our survey.

**Section A:** Consent

1. Do you agree that any personal information provided in this survey can be used for study purposes?

[1] Yes [2] No *(end of survey)*

**Section B:** Demographics characteristics

1. What is your age?

2. What is your gender?

[1] Male [2] female [3] others

3. What is your marital status?

[1] Single [2] Marriage [3] Separated [4] Widowed [5] Divorced

4. What is your religion?

[1] Buddhist [2] Christianity [3] Muslim

**Section C:** Socio-economic factors

1. What is your occupation?

[1] Doctors [2] Dentists [3] Pharmacist [4] Nurses

[5] Medical laboratories [6] Patient aids

[7] Village health volunteers and migrant health volunteers

[8] Public health officers [9] Others

2. What is your province of work?

3. Which types of your work area?

[1] Urban area [2] Rural area

4. Do you have more than one workplace?

[1] Yes [2] No

5. What type of workplace that you spend the majority of your time?

[1] Public health centers [2] Community hospitals

[3] General hospitals [4] Specialized hospitals

[5] Teaching hospitals [6] District/provincial health offices

[7] Ministry of Public Health [8] Private hospitals/clinics

[9] Pharmacies [10] Other health facilities (Retired or no longer working)

6. Do you are a frontline COVID-19 worker?

[1] Yes [2] No

**Section D:**  Health-related factors

1. Do you have any health conditions? *(can chooses multiple answers)*

[1] Diabetes [2] Cardiovascular diseases [3] Kidney diseases

[4] Cancer [5] Obesity (BMI ≥ 25 kg/m^2^) [6] Respiratory diseases

[7] Others

2. Have you received Influenza vaccines before?

[1] Had [2] Never had [3] Uncertain

3. Had COVID-19 infection?

[1] Had [2] Never had [3] Uncertain

**Section E:** Perceived risk for COVID-19

1. Had direct contact in the screening of individuals at risk for COVID-19 or the caring of COVID-19 patients?

[1] Yes [2] No

2. How do you perceive the risk of COVID-19?

[1] No risk [2] Low risk [3] Moderately low risk

[4] Moderate risk [5] Moderately high risk [6] High risk

3. Whom are you living with as the risk of COVID-19 conditions?

[1] Living with older adults (≥ 60 years)

[2] Living with chronic patients

[3] Living with both older adults and chronic patients

[4] Living with neither older adults nor chronic patients

*Note: Chronic diseases are diabetes mellitus, hypertension, stroke, coronary artery disease, cancer with chemotherapy treatment, chronic obstructive pulmonary disease, stage 5 kidney disease, obesity or BMI ≥ 25 kg/m^2^*

4. What is your location perceived to be the highest risk of COVID-19 infection?

[1] Home [2] Community [3] Workplace

**Section F**: Perceived enablers and barriers of COVID-19 vaccination

1. How many doses of the COVID-19 vaccine did you get?

[1] None [2] A dose *(skip to question 3^rd^ )* [3] Two doses *(skip to question 4^th^)*

2. Would you be willing to receive the COVID-19 vaccine?

[1] Yes [2] No [3] Uncertain

3. Would you be willing to receive the second dose of the COVID-19 vaccine?

[1] Yes [2] No [3] Uncertain

4. Which is condition(s) support you to accept the vaccine?

[1] If they received the vaccine they prefer (based on the list of existing vaccines regardless of availability in Thailand)

[2] If a certain number of people were vaccinated in the world without serious adverse events

5. What are the top three enablers to receive the COVID-19 vaccine?

[1] If WHO or FDA in Thailand recommended COVID-19 vaccination

[2] If your family or friends received the COVID-19 vaccine

[3] Believe that the COVID-19 vaccine will stop infection

[4] Believe that the COVID-19 vaccine will prevent transmission

[5] Believe that the COVID-19 vaccine will prevent mortality

[6] You have some condition(s) health

[7] Your occupation has a high risk of COVID-19 infection

[8] You live in an area that a high risk of COVID-19 infection

6. What are the top three barriers to receiving the COVID-19 vaccine?

[1] Concern about the efficacy of COVID-19 vaccination

[2] Concern about the short-term side effect of COVID-19 vaccination

[3] Concern about the long-term side effect of COVID-19 vaccination

[4] You don't contact with COVID-19 cases

[5] Transportation barriers to getting the COVID-19 vaccine

[6] Travel restriction

[7] Cannot receive the COVID-19 vaccine, e.g., pregnancy, blood clotting disorders?

[8] Low risk of death from the COVID-19 infection

7. Would you recommend others to receive the COVID-19 vaccine?

[1] Yes [2] No [3] Uncertain
